# Early Predictors of School Absenteeism in First-Grade Children: A Multidimensional Longitudinal Study in Japan

**DOI:** 10.3390/children12091265

**Published:** 2025-09-20

**Authors:** Yuki Soma, Yu Ogasawara, Hiromi Kobayashi

**Affiliations:** 1 Faculty of Education, Hirosaki University, Hirosaki 036-8560, Japan; 2 Faculty of Urban Environmental Sciences, Tokyo Metropolitan University, Hachioji 192-0397, Japan; 3 Graduate School of Education, Hirosaki University, Hirosaki 036-8560, Japan

**Keywords:** absenteeism, school health room, academic performance, lifestyle, first-grade students

## Abstract

**Highlights:**

**What are the main findings?**

- Academic performance and the frequency of school health room use due to illness are common predictors of school absenteeism in both boys and girls.
- Fewer days of physical activity and poor sleep quality were additional predictors of school absenteeism in girls.

**What is the implication of the main finding?**

- First-grade elementary school students who exhibit risk factors should be carefully monitored and supported in dealing with school difficulties.
- Academic and psychological support provided by school staff is an effective way to prevent absenteeism.

**Abstract:**

**Background**: Continuous schooling and healthy learning are essential during childhood. Therefore, we investigated the risk factors of absenteeism in supporting at-risk students. The aim of this study examined the longitudinal relationships between absenteeism and body mass index (BMI), lifestyle, physical fitness, academic performance, and frequency of school health room use during the first grade of elementary school. **Methods**: A total of 453 first-grade students in elementary school were included in the analysis. These students were enrolled in the target elementary schools between 2014 and 2021. The analysis used data obtained from the students’ schoolwork. We performed a Cox regression analysis to assess the characteristics associated with absenteeism for 10 days or more, excluding absences due to relatives’ funerals and suspension of attendance. The independent variables were BMI, sleep duration, sleep quality, frequency of physical activity, screen time, physical fitness, grade points, and frequency of health room use during the first grade of elementary school. **Results**: The grade point average (girls: hazard ratio [HR] = 0.013; boys: HR = 0.115) and frequency of use of school health rooms due to illness (girls: HR = 1.252; boys: HR = 1.261) were common among girls and boys in the adjusted model. Additionally, three or more days of physical activity per week and six or more days of sound sleep were additional predictors for girls. **Conclusions**: Our results suggest that careful monitoring of children with minimal physical activity, poor sleep quality, low grade point average, and frequent school health room usage in the first grade of elementary school and providing them with support in dealing with school difficulties may reduce absenteeism.

## 1. Introduction

An individual’s intelligence level and educational attainment influence chronic disease prevalence [1,2,3], life expectancy [4], disability-free life expectancy [5], and child mortality [6]. Therefore, continuous education and healthy learning are crucial during childhood. However, high absenteeism among students poses several challenges for various reasons [7]. In particular, school absences lead to a loss of educational opportunities. Several studies on the risk factors for absenteeism have been conducted worldwide to support at-risk students.

Absenteeism refers to excused or unexcused absences related to medical illnesses, injuries, environmental, social, psychiatric, and other factors [7]. The criteria for absenteeism vary by country, region, and research context, with a reported range of 10–30 school days absent per year [8,9,10]. Many studies have examined the correlates of school absenteeism worldwide, identifying the following factors: obesity (or being overweight) [11,12,13], inadequate sleep [14,15], physical activity [16], prolonged screen time and sedentary behavior [12], low physical fitness [17], and poor academic performance [18,19].

Overweight and obesity have been frequently linked to school absenteeism. According to a study conducted in students aged 6–13 years and 14–17 years in Australia, obese children were absent for 1.07 days and 0.69 days more, respectively, than healthy-weight children, indicating a higher risk of absenteeism [11]. Additionally, a study of 11–17-year-old children in 71 countries found that overweight and obese children were 1.11 and 1.17 times more likely to miss school for 3 days or more for any reason during the past 30 days, respectively, than healthy-weight children [12]. While some studies have reported no association between school attendance and body mass index (BMI) [18], a meta-analysis by An et al. showed that obese and overweight children have 27% and 54% higher risks of school absenteeism, respectively, than normal-weight children [13].

Moreover, lifestyle factors, such as sleep and physical activity, have been reported to contribute to children’s school absenteeism. A study on Norwegian adolescents aged 16–19 years found that short sleep duration, insomnia, and a delayed sleep-wake phase were associated with an increased risk of school dropout [14], with each additional hour of sleep decreasing the number of absences by 6% [15]. Physical activity habits, such as screen time and amount of exercise, were examined. Hansen et al. indicated that watching television for more than two hours daily was positively associated with school absenteeism [16]. In contrast, some studies have reported a U-shaped association between physical activity and absenteeism. Inactive and highly active children were at higher risk of school absenteeism than moderately active children [16]. Long sitting times have also been reported as a risk factor for absenteeism [12]. However, the results of studies utilizing accelerometers have been inconsistent [20,21]. Although several studies have indicated an association between physical activity and absenteeism, research on their relationship is limited. One study indicated a positive correlation between low physical fitness and an increased risk of absenteeism [17].

Academic performance is also an important factor in absenteeism. In a study on Turkish high school students, a structural equation model revealed that poor past academic performance was associated with current absenteeism [19]. Similarly, a study of fourth-grade children in Colombia reported a negative association between academic performance and the number of days absent [18]. These studies suggest that providing academic support for low-achieving students may be an effective strategy for preventing absenteeism [22].

Although various studies on the causes of school absenteeism have been conducted worldwide, research in Asian countries is limited [23]. Furthermore, although breakfast habits have been suggested as a factor affecting children’s academic performance [24], their relationship has not been explored in a study. Additionally, school health rooms provide health care to children with physical or mental health issues. In Japan, school health rooms are managed by teachers called “Yogo teachers,” known as “school nurses” in other countries [25]. “Yogo teacher” refers to a unique school staff position in Japan: a full-time teacher who provides physical and mental support to students through health counseling, minor injury care, health education, conducting health checkups, and providing recommendations to parents for medical visits. They assisted in monitoring the use of school health rooms by recording the treatments administered to students. School health rooms serve as safety nets for students at high risk of absenteeism. Therefore, it is plausible that the risk of absenteeism can be identified using school health records. However, no previous studies have examined the relationship between school health room use and future school absenteeism using follow-up data.

This study aimed to examine the longitudinal relationship between school absenteeism and BMI, sleep, physical activity, screen time, physical fitness, academic performance, and school health room records during the first grade of elementary school. To achieve this aim, we analyzed accumulated data of elementary and junior high school students in Japan.

## 2. Materials and Methods

### 2.1. Study Design and Population

This retrospective cohort study aimed to explore factors associated with school absenteeism. To achieve this aim, data on absenteeism and its associated factors were collected from targeted schools from 2014–2021. Data availability is summarized in Table 1. Using these data, we analyzed the factors associated with 10 or more days of absence during the 1–7-year follow-up period among first-grade elementary school children using a survival analysis.

This study targeted elementary and junior high schools in a rural area of Hirosaki City, Aomori Prefecture, located 550 km north of Tokyo in a high snowfall region of Japan. The two schools registered 909 students in September 2022. A total of 453 first-grade elementary school students were included in the analysis, excluding those whose parents declined to provide data (n = 28), those with incomplete research IDs (n = 1), and those with no school attendance data in the first grade of elementary school (n = 306) because they enrolled in the second grade or later. Students were also excluded in cases where continuous follow-up was not possible because of missing data or students transferring in or out of the school (n = 6), as well as in cases with missing data (n = 115). Most of the targeted elementary school students entered the target junior high school.

### 2.2. Data Collection

Secondary data collected from the school activities was extracted and analyzed. The data involved students enrolled in the targeted schools as of 2022. These schools digitized and anonymized the data before sharing it with the researchers. The data used for the analysis are as follows:

#### 2.2.1. Days of Absence from School

School attendance was recorded at the end of each school year. The number of days absent from school, excluding absences due to relative funerals and designated infectious diseases (including COVID-19 and related absences), was extracted for analysis. Absence was defined as not attending school for the entire school day and was therefore distinguished from tardiness or early departure. The analysis considered days absent from the first grade of elementary school until the end of the follow-up period (1–7 years).

#### 2.2.2. Height, Weight, and BMI

In the first grade of elementary school, height and weight were measured at the beginning of the school year (generally from April–May) according to the Health Checkup Manual for School Children [26]. The BMI was calculated based on the height and weight [BMI = Weight (kg)/Height (m)^2^].

#### 2.2.3. Sleep, Physical Activity, Screen Time, and Breakfast Habits

A survey on sleep, physical activity, screen time, and breakfast habits was conducted from May–July, following the Physique, Fitness, and Lifestyle Survey Guidelines [27]. The survey targeted students who completed it with the support of their teachers. It included the following items: sleep duration (<6 h/day, 6–8 h/day, or ≥8 h/day); number of days with sound sleep (≥6 days/week, 4–5 days/week, 2–3 days/week, or <1 day/week); physical activity outside of physical education classes (≥3 days/week, 1–2 days/week, 1–3 days/month, or never); screen time (<1 h/day, 1–2 h/day, 2–3 h/day, or ≥3 h/day); and breakfast habits (every day, sometimes, or never). These are standard survey questions used in Japanese schools to examine children’s lifestyle habits. For those with unavailable data from the first year of elementary school, we imputed data from the second year (n = 78).

#### 2.2.4. Physical Fitness

At the beginning of the school year (generally, May–July), first-grade elementary students’ muscle strength [grip strength (kg)], flexibility [sit and reach (cm)], agility [sidestep (times/20 s)], cardiovascular endurance [20 m shuttle run (times)], muscular speed [50 m sprint (sec)], and muscular power [standing long jump (cm) and softball throw (m)] were measured according to the Japan Fitness Test manual [28]. These are standard tests used in schools in Japan to safely assess children’s physical fitness.

#### 2.2.5. Academic Performance

The academic performance of first-grade elementary school students in Japanese language, arithmetic, living environment studies, music, art and handicrafts, and physical education was evaluated at the end of the school year. Teachers considered various criteria in their evaluations, including “interest, eagerness, and attitude,” “ability in respective subjects (e.g., numbers and calculations, quantities, and measurements, geometrical figures, and mathematical relations in arithmetic),” and “knowledge, understanding, and skills.” Starting in 2020, all subjects were evaluated based on the following items: “fundamental knowledge and skills,” “ability to think, to judge, to express oneself,” and “an attitude of proactive learning.” Each item was rated on a three-point scale (A, B, and C, in order of excellence). Each rating was converted to a numerical score, i.e., three points for A, two for B, and one for C. For quantitative analysis, each student’s item scores were averaged and used as their grade point (GP).

#### 2.2.6. Frequency of School Health Room Use

The frequency of school health room use for injuries and illnesses in the first grade of elementary school during the year was obtained from school health room records. The total frequency was calculated by summing the numbers of injuries and illnesses.

### 2.3. Statistical Analysis

We calculated the numbers and percentages (%) for categorical variables and the median, first quartile, and third quartile for continuous variables.

In Japan, school absence refers to being absent from school for more than 30 days. However, since only 10 students were absent for more than 30 days, and the previous study used 10 days as the cutoff, the present study also used an absence of 10 or more days as the outcome [29]. Cox regression analysis was used to examine the association between 10 or more days of absenteeism and BMI, sleep, physical activity, screen time, physical fitness, academic performance, and frequency of school health room use. Hazard ratios (HRs) and 95% confidence intervals (95% CIs) were calculated by Cox regression analysis. Survival curves for each dichotomous variable were plotted using the Kaplan–Meier method, and differences between groups were assessed using the log-rank test. The grade point average (GPA) was calculated as the average of the GPs for all students. Study variables were dichotomized as follows: sleep duration (0: <8 h/day; 1: ≥8 h/day); number of days with sound sleep (0: <6 days/week; 1: ≥6 days/week); physical activity (0: <3 days/week; 1: ≥3 days/week); screen time (0: ≥2 h/day; 1: <2 h/day); and breakfast habits (0: Not daily; 1: daily). Variables with a significance probability of less than 10% (*p* < 0.10) in the crude model were included in the multivariate model to examine the independent associations.

The data were analyzed using R software (Version 4.3.1) with skimr (Version 2.1.5) and survival packages (Version 3.5–7). The significance level was set at less than 5% (*p* < 0.05).

## 3. Results

### 3.1. Student Characteristics

**Figure 1:**
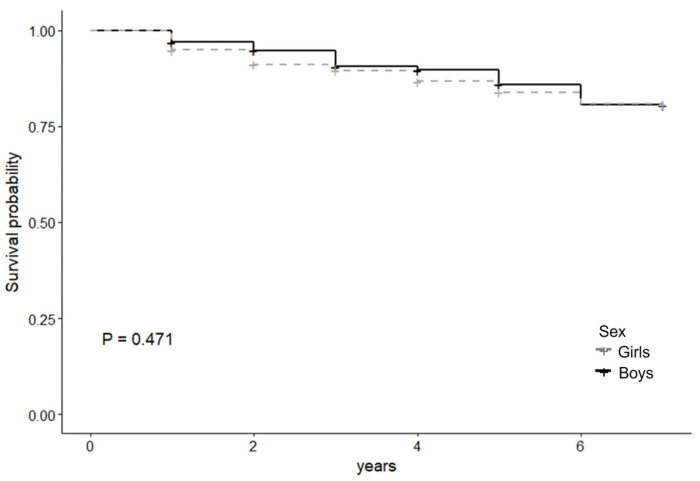
Survival curve by gender. *p*-value based on the log-rank test.

Table 2 shows the characteristics of the analyzed population according to sex. Among the students, 51% were girls, and 50 students (27 girls, 11.7%; 23 boys, 10.4%) were absent for 10 days or more. The median follow-up period was three years (first–third quartiles: 2–5 years) for both girls and boys. Figure 1 and Table 3 show the survival curves and frequency of 10 or more days of school absenteeism by sex, respectively. Absenteeism of 10 days or more per year did not occur in a specific school year, but at specific rates throughout the follow-up period.

### 3.2. The Correlates of Absenteeism

**Figure 2:**
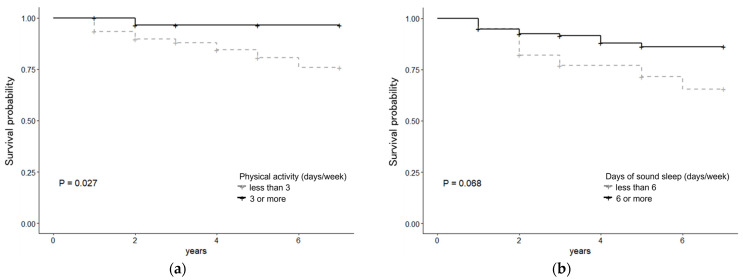
Survival curves for (**a**) physical activity and (**b**) sound sleep among girls. *p*-value based on the log-rank test.

Table 4 presents the correlations of absenteeism among the girls. Regarding physical fitness, the HR for long-standing jumps was lower (HR 0.975, 95% CI, 0.952–0.999). Higher grades in Japanese Language (HR 0.119, 95%CI 0.041–0.347), Living Environment Studies (HR 0.094, 95%CI 0.022–0.411), Music (HR 0.228, 95%CI 0.064–0.817), Arts and Handicraft (HR 0.191, 95%CI 0.046–0.795), Physical Education (HR 0.227, 95%CI 0.057–0.900), and GPA (HR 0.025, 95%CI 0.004–0.156) were negatively correlated with absenteeism for 10 days or more. A high HR was found for frequent use of the school health room (HR 1.096, 95% CI, 1.020–1.179) and a higher ratio for frequency due to illness (HR 1.156, 95% CI, 1.060–1.261). Six or more days of sound sleep, three or more days a week of physical activity, grip strength, and good arithmetic GP were among the variables with significant trends (*p* < 0.10). Figure 2 illustrates the survival curve for the frequency of physical activity and days of sound sleep among girls. A significant difference was observed between physical activity for three or more days and that for less than three days. As academic performance and frequency of use of the school health room were considered highly collinear in the multivariate model, the GPA and frequency of use of the school health room due to illness were entered as representative values. Multivariate analysis revealed significant and independent associations between six or more days of sound sleep (HR 0.181, 95%CI 0.069–0.473), three or more days per week of physical activity (HR 0.047, 95%CI 0.006–0.373), GPA (HR 0.013, 95% 0.002–0.112), frequency of use of the school health room due to illness (HR 1.252, 95%CI 1.145–1.368), and absenteeism among girls.

Table 5 shows the correlation between absenteeism and boys. Higher grades in Music (HR 0.213, 95%CI 0.053–0.862) and GPA (HR 0.137, 95%CI 0.027–0.690) were negatively correlated with absenteeism for 10 days or more. The hazard ratios for the frequency of use of the school health room were higher due to illness (HR 1.227, 95%CI 1.053–1.431). Moreover, the multivariate analysis revealed significant and independent associations between GPA (HR 0.115, 95%CI 0.022–0.588), frequency of use of the school health room due to illness (HR 1.261, 95%CI 1.076–1.478), and absenteeism among boys.

## 4. Discussion

This study analyzed data on BMI, lifestyle, physical fitness, academic performance, and frequency of school health room use in the first grade of elementary school to identify predictors of 10 or more days of absenteeism. The results showed that academic performance and use of school health rooms due to illness were common predictors for both girls and boys in the adjusted model, with sleep quality and physical activity being additional predictors for girls.

This study is the first to determine whether the frequency of school health room use due to illness is a predictor of absenteeism. We found that the risk of absenteeism increased by a multiplicative factor of 1.2 for each visit to the school health room. Students use the school health room because of illness symptoms, such as headaches, stomachaches, and feeling ill [30,31]. These symptoms can also be caused by stress (any adverse stimulus, physical, mental, emotional, etc.) [32]. As stress is a factor that causes repeated absence due to illness, the frequency of school health room use due to illness is considered a precursor for absenteeism. Therefore, Yogo teachers, school counselors, and parents must work together to establish a support system for students visiting school health rooms due to illnesses.

Moreover, academic performance was considered a predictor of 10 days or more of absenteeism. The results of this study are consistent with those of previous studies [18,19]. In this study, students’ academic performance was subjectively assessed by schoolteachers. Similar results were obtained in previous studies; however, this assessment was less objective than that of the previous study based on national standardized achievement test scores. Sitting in a classroom for a prolonged period without genuine learning or understanding of the lessons is a concern for students. Poor academic performance is associated with low satisfaction with classes and schools [33,34], which may lead to mental health problems and absenteeism. Studies have shown that supplemental academic training, such as remedial education, tutoring, and homework assistance, can prevent absenteeism due to a lack of understanding of class content [22].

At least three days of physical activity per week in the first grade was associated with reduced absenteeism among girls. The results of this study align with those of previous studies that state that physical activity reduces absenteeism [16]. However, contrary to the findings of previous studies, this study did not find screen time to be a significant predictor [16]. Furthermore, prior research has indicated that poor physical ability may lead to bullying. In particular, it has been emphasized that girls may be forced to participate in competitive games during physical education classes, which can lead to negative experiences such as teasing and social exclusion by their peers [35]. The crude model demonstrated that the GP of physical education and standing long jump were significant predictors of 10 days or more of absenteeism per year in girls, alongside physical activity, suggesting that students who are not physically active and fit have high absenteeism. Students’ unfavorable perceptions of physical activity may have contributed to their frequent absenteeism. Hansen et al. stated that while watching TV is associated with absenteeism, computer usage time is not [16]. Therefore, screen time was not considered a significant predictor of absenteeism, as it also included time spent playing games. This suggests that passive and inactive screen time influences absenteeism. From a mental health perspective, physical activity impacts girls and boys similarly [36]. However, girls tend to report poorer mental health than boys [37,38]. Hence, physical activity may have a greater effect on improving the mental health of girls. This could explain why a significant association was observed among girls. Thus, supporting girls with inactive lifestyles and difficulties with physical education in the first grade of elementary school is essential. Additionally, class content and teaching methods that support girls and help them adapt to their inability to perform tasks in physical education classes are necessary.

The results also showed that sleep quality was a more significant predictor of sleep habits than sleep duration. Absenteeism occurred less frequently in patients with better sleep quality. Sleep is also associated with mental health in children [39,40]. Our findings regarding the relationship between absenteeism and sleep are consistent with those of previous studies. Similar to the impact of physical activity on girls’ mental health, the impact of sleep quality may be greater in girls than in boys [38]. Therefore, sleep quality instead of duration should be prioritized over time to maintain and improve children’s health [41]. This indicates that health education programs should focus on sleep duration and quality.

In the present study, BMI was not associated with 10 days or more of absenteeism per year. One reason for this is that the BMI of the study population was lower than that of children of the same age in other countries. Children of the same ages in large-scale studies conducted in the Netherlands, the United Kingdom, and the United States had average BMI values of 16.09, 16.54, and 16.99, respectively [42]. These values are higher than the median study value of 15.4 and lower than those reported in other European countries [43], indicating a notably lower prevalence of obesity in the study sample. Furthermore, because the targeted schools require entrance exams, they tend to enroll children with higher literacy levels. This may make obese children less likely to be targets of teasing or bullying.

This study examined schools in rural areas with heavy snowfall. People in rural areas generally have lower educational attainment and socioeconomic status. Both these factors have been reported to be associated with school absenteeism [44]. Therefore, children in the targeted schools present a high-risk group for absenteeism compared with children in urban areas. Consequently, the findings of this study can only be generalized to children in rural areas of a similar size.

This study had several limitations. The results were obtained from elementary and junior high schools in a medium-sized city in a high-snowfall region of Japan. Therefore, generalizations to other contexts should be made with caution. In particular, compared to students in other countries, Japanese students have fewer days of absence. This is thought to stem from the Confucian welfare system, comprehensive education system, and Japanese values that place lower value on individualism and higher value on uncertainty [45]. Second, this study focused on students with high academic abilities who were enrolled in schools that selected them for admission. This population was considered to have a lower risk of absenteeism than that of previous studies. Hence, the associations between absenteeism and the factors of interest may have been underestimated. Third, this study did not identify the reasons for absenteeism or categorize cases of absenteeism as school refusal, illness, or truancy. Fourth, academic performance was subjectively evaluated from the perspective of classroom teachers instead of using a more objective assessment, such as national standardized test scores. Although scores from the end-of-unit tests are reflected in teachers’ assessments, there were no clear evaluation criteria; therefore, the final grade was left to the teacher’s discretion. Furthermore, reverse causality may be occurring, whereby academic performance is reflected in attendance records. Fifth, this retrospective study may have missed significant exposures related to school absenteeism. In particular, the family and peer domains have been reported as predictors of children’s school absenteeism and academic performance [44,46]. These factors have not been controlled in the present study. Furthermore, because secondary data were collected for purposes other than the present study, school records may be incomplete. However, schools that use examination-based selection for admission, such as the target schools in this study, include students with specific levels of academic achievement and life skills. Therefore, socioeconomic factors have little influence on our results. Sixth, the World Health Organization recommends an average of 60 min of moderate-to-vigorous physical activity per day [47], and the Sleep Guide for Health Promotion 2023 in Japan recommends 9–12 h of sleep per day for elementary school students [48]. However, participants in this study reported at most “≥3 days/week” of exercise and sleep durations of “≥8 h/day.” Hence, the recommended time-based criteria could not be applied. Finally, sleep duration, physical activity, and breakfast consumption data were obtained from subjective responses that were not validated. Therefore, it is possible that respondents exhibited information bias by attempting to provide answers that were generally considered desirable. In future studies, the use of activity monitors that objectively measure physical activity and sleep habits is recommended. Despite these limitations, the study findings are reliable, as they analyzed seven years of longitudinal data to identify the factors predictive of absenteeism for 10 days or more. Furthermore, our findings indicated that the frequency of school health room use for illness was a significant predictor of the outcome. Therefore, our findings have practical implications and can be used to develop methods to help students achieve healthy school lives.

## 5. Conclusions

This study examined the predictors of frequent absenteeism (at least 10 days per year) among elementary and junior high school students, excluding absences owing to unavoidable reasons, such as infectious diseases or relatives’ funerals. The results showed that frequent use of school health rooms due to illness and poor academic performance were predictive factors for both girls and boys. Moreover, poor sleep quality and three days of physical activity per week among girls were also associated with absenteeism. Therefore, to address absenteeism, it is necessary to carefully monitor children who exhibit these factors in the first grade of elementary school and support them in dealing with school difficulties. The findings of this study can be used to identify children at high risk of school absenteeism by the first grade and to implement interventions. For example, effective interventions for both girls and boys could include academic support from teachers and learning support staff, as well as psychological support from Yogo teachers and school counselors. In addition to these interventions, girls should be provided with guidance on lifestyle improvements. The analytical procedures used in this study are also effective for exploring school-specific factors contributing to school absenteeism. Therefore, it is desirable to develop guidelines that outline how to utilize the results derived from these analytical procedures. However, this study had a significant limitation in that it used data from a single school in one region. Therefore, a multi-school study encompassing various regions is necessary to expand this research.

## Figures and Tables

**Table 1:** Data availability for each grade.

		Grades for Which Data are Available						
		1st	2nd	3rd	4th	5th	6th	7th
Grade in 2022	1st							
	2nd	†						
	3rd	O	†					
	4th	O	O	†				
	5th	O	O	O	†			
	6th	O	O	O	O	†		
	7th ^1^	O	O	O	O	O	†	
	8th ^2^	O	O	O	O	O	O	O
	9th ^3^	O	O	O	O	O	O	O

O: Available, Blank: Not available. ^1^ 7th: 1st grade in junior high school, ^2^ 8th: 2nd grade in junior high school, ^3^ 9th: 3rd grade in junior high school. † Due to data linkage issues, data on days of absence from elementary schools in 2021 could not be collected.

**Table 2:** Student characteristics in present study.

		Overall	Girls	Boys
		n = 453	n = 231	n=222
Girls		231 (51.0)		
Follow-up years	Years	3 (2–5)	3 (2–5)	3 (2–5)
School absenteeism	≥10 days/year	50 (11.0)	27 (11.7)	23 (10.4)
Height	cm	116.8 (113.5–120.0)	116.2 (113.2–119.4)	117.5 (114.0–120.5)
Weight	kg	20.9 (19.2–23.2)	20.4 (19.1–22.8)	21.3 (19.3–23.8)
Body Mass Index	kg/m^2^	15.4 (14.5–16.5)	15.3 (14.4–16.3)	15.5 (14.6–16.8)
Sleep duration	<6 h/day	24 (5.3)	14 (6.1)	10 (4.5)
	6–8 h/day	91 (20.1)	56 (24.2)	35 (15.8)
	≥8 h/day	338 (74.6)	161 (69.7)	177 (79.7)
Days of sound sleep	≥6 days/week	369 (81.5)	191 (82.7)	178 (80.2)
	4–5 days/week	43 (9.5)	22 (9.5)	21 (9.5)
	2–3 days/week	27 (6.0)	12 (5.2)	15 (6.8)
	<1 day/week	14 (3.1)	6 (2.6)	8 (3.6)
Physical activity	≥3 days/week	110 (24.3)	49 (21.2)	61 (27.5)
	1–2 days/week	256 (56.5)	135 (58.4)	121 (54.5)
	1–3 days/month	59 (13.0)	33 (14.3)	26 (11.7)
	Never	28 (6.2)	14 (6.1)	14 (6.3)
Screen time	<1 h/day	151 (33.3)	86 (37.2)	65 (29.3)
	1–2 h/day	181 (40.0)	87 (37.7)	94 (42.3)
	2–3 h/day	87 (19.2)	41 (17.7)	46 (20.7)
	≥3 h/day	34 (7.5)	17 (7.4)	17 (7.7)
Breakfast	Every day	428 (94.5)	219 (94.8)	209 (94.1)
	Sometimes	24 (5.3)	11 (4.8)	13 (5.9)
	Never	1 (0.2)	1 (0.4)	0 (0.0)
20 m shuttle run	times	12 (9–17)	11 (9–15)	14 (10–18)
50 m sprint	sec	12.1 (11.5–13.0)	12.3 (11.7–13.2)	12 (11.3–12.7)
Grip strength	kg	8 (7–10)	8 (6–9)	9 (7–10)
Standing long jump	cm	105 (95–117)	102 (93–115)	110 (100–120)
Sidestep	times	27 (24–29)	26 (24–29)	27.5 (25–30)
Sit and reach	cm	27 (23–31)	28 (24–32)	26 (21–30)
Softball throw	m	6 (5–8)	5 (4–6)	7 (6–10)
Japanese language	GP	2.6 (2.4–2.8)	2.8 (2.4–3.0)	2.6 (2.2–2.8)
Arithmetic	GP	2.8 (2.5–3.0)	2.8 (2.5–3.0)	2.8 (2.5–3.0)
Living environment Studies	GP	2.7 (2.5–3.0)	2.7 (2.7–3.0)	2.7 (2.3–2.7)
Music	GP	2.8 (2.5–2.8)	2.8 (2.5–3.0)	2.5 (2.3–2.8)
Art and handicraft	GP	2.5 (2.3–2.8)	2.7 (2.5–2.8)	2.5 (2.3–2.8)
Physical education	GP	2.5 (2.3–2.7)	2.3 (2.3–2.7)	2.5 (2.3–2.7)
GPA	GP	2.6 (2.5–2.8)	2.7 (2.5–2.8)	2.6 (2.4–2.7)
Use of school health room	times/year	3 (1–6)	3 (1–6)	3 (1.3–6)
Illness	times/year	1 (0–2)	1 (0–2)	1 (0–2)
Injury	times/year	2 (1–4)	1 (0–3)	2 (1–4)

Categorical variables are presented as n (%) and quantitative variables as median (1st–3rd quartile). GP: grade point, GPA: grade point average.

**Table 3:** Number of occurrences of ten or more days of school absenteeism.

Follow-Up Year	1	2	3	4	5	6	7
Overall	19	11	8	4	5	3	0
Boys	7	4	6	1	3	2	0
Girls	12	7	2	3	2	1	0

**Table 4:** HRs of incident 10 or more days of absenteeism in girls.

	Crude Model		Adjusted Model	
	HR (Lower–Upper)		HR(Lower–Upper)	
BMI	1.034 (0.825–1.295)			
Sleep duration (≥8 h/day)	0.820 (0.367–1.830)			
Days of sound sleep (≥6 days/week)	0.467 (0.202–1.081)	†	0.181 (0.069–0.473)	*
Physical activity (≥3 days/week)	0.145 (0.020–1.072)	†	0.047 (0.006–0.373)	*
Screen time (<2 h/day)	0.683 (0.306–1.523)			
Breakfast (Everyday)	0.490 (0.147–1.632)			
20 m shuttle run	0.940 (0.861–1.026)			
50 m sprint	1.260 (0.952–1.668)			
Grip strength	0.838 (0.683–1.029)	†	0.902 (0.720–1.130)	
Standing long jump	0.975 (0.952–0.999)	*	0.986 (0.961–1.012)	
Sidestep	0.956 (0.889–1.027)			
Sit and reach	0.979 (0.925–1.035)			
Softball throw	0.862 (0.688–1.079)			
Japanese Language GP	0.119 (0.041–0.347)	*		
Arithmetic GP	0.426 (0.165–1.099)	†		
Living Environment Studies GP	0.094 (0.022–0.411)	*		
Music GP	0.228 (0.064–0.817)	*		
Art and Handicraft GP	0.191 (0.046–0.795)	*		
Physical Education GP	0.227 (0.057–0.900)	*		
GPA	0.025 (0.004–0.156)	*	0.013 (0.002–0.112)	*
Use of school health room (total)	1.096 (1.020–1.179)	*		
Use of school health room (illness)	1.156 (1.060–1.261)	*	1.252 (1.145–1.368)	*
Use of school health room (injury)	1.049 (0.904–1.217)			

The HR and 95% confidence interval are presented. GP: grade point, * *p* < 0.05, † *p* < 0.10. All variables with a significance probability of less than 10% in the crude model were included in the adjusted model.

**Table 5:** HRs of incident 10 or more days of absenteeism in boys.

	Crude Model		Adjusted Model	
	HR (Lower–Upper)		HR (Lower–Upper)	
BMI	1.122 (0.909–1.386)			
Sleep duration (≥8 h/day)	0.895 (0.328–2.441)			
Days of sound sleep (≥6 days/week)	1.002 (0.362–2.776)			
Physical activity (≥3 days/week)	1.754 (0.742–4.148)			
Screen time (<2 h/day)	1.372 (0.509–3.698)			
Breakfast (Everyday)	1.674 (0.224–12.530)			
20 m shuttle run	0.975 (0.915–1.040)			
50 m sprint	1.058 (0.775–1.445)			
Grip strength	1.072 (0.872–1.318)			
Standing long jump	0.996 (0.972–1.019)			
Sidestep	1.041 (0.974–1.113)			
Sit and reach	0.978 (0.921–1.038)			
Softball throw	0.947 (0.824–1.088)			
Japanese Language GP	0.423 (0.162–1.106)	†		
Arithmetic GP	0.383 (0.143–1.025)	†		
Living Environment Studies GP	0.338 (0.105–1.089)	†		
Music GP	0.213 (0.053–0.862)	*		
Art and Handicraft GP	0.319 (0.078–1.300)			
Physical Education GP	0.543 (0.155–1.903)			
GPA	0.137 (0.027–0.690)	*	0.115 (0.022–0.588)	*
Use of school health room (total)	1.050 (0.953–1.157)			
Use of school health room (illness)	1.227 (1.053–1.431)	*	1.261 (1.076–1.478)	*
Use of school health room (injury)	0.980 (0.841–1.142)			

The HR and 95% confidence interval are presented. GP: grade point, * *p* < 0.05, † *p* < 0.10. All variables with a significance probability of less than 10% in the crude model were included in the adjusted model.

## Data Availability

The datasets generated and analyzed in this study are not publicly available because of ethical restrictions. This study collected potentially sensitive information from students under the age of 18 years, and the publication of the datasets and provision to third parties were not covered under ethical approval.

## Abbreviations

The following abbreviations are used in this manuscript:

HR	Hazard Ratio
GP	Grade Point
GPA	Grade Point Average
BMI	Body Mass Index
CI	Confidence Interval
